# Potential of Biological Agents in Decontamination of Agricultural Soil

**DOI:** 10.1155/2016/1598325

**Published:** 2016-05-03

**Authors:** Muhammad Kashif Javaid, Mehrban Ashiq, Muhammad Tahir

**Affiliations:** Department of Chemistry, University of Gujrat, Gujrat 50700, Pakistan

## Abstract

Pesticides are widely used for the control of weeds, diseases, and pests of cultivated plants all over the world, mainly since the period after the Second World War. The use of pesticides is very extensive to control harm of pests all over the globe. Persistent nature of most of the synthetic pesticides causes serious environmental concerns. Decontamination of these hazardous chemicals is very essential. This review paper elaborates the potential of various biological agents in decontamination of agricultural soils. The agricultural crop fields are contaminated by the periodic applications of pesticides. Biodegradation is an ecofriendly, cost-effective, highly efficient approach compared to the physical and chemical methods which are expensive as well as unfriendly towards environment. Biodegradation is sensitive to the concentration levels of hydrogen peroxide and nitrogen along with microbial community, temperature, and pH changes. Experimental work for optimum conditions at lab scale can provide very fruitful results about specific bacterial, fungal strains. This study revealed an upper hand of bioremediation over physicochemical approaches. Further studies should be carried out to understand mechanisms of biotransformation.

## 1. Introduction

**Figure 1:**
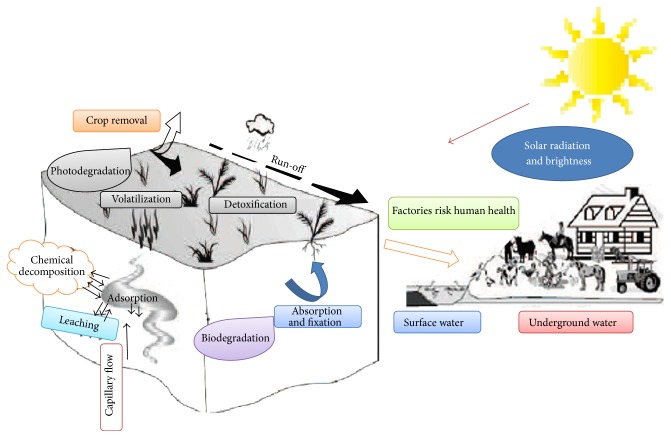
Pesticides contaminant and biodegradation in environment [11, 12].

A pesticide can be defined as any substance or mixture of substances intended for preventing, destroying, repelling, or mitigating any pest (insects, mites, nematodes, weeds, rats, etc.). Pesticides like insecticides, herbicides, fungicides, and various other substances are used to control pests [1]. In modern agriculture practices, the extensive use of pesticides is very frequent to fulfill higher yield requirements. Millions of tons of pesticides are applied annually all over the globe, which covers the billions of dollars' market. The expenditures on pesticides were 35.8 billion in 2006 which rose up to 39.4 billion US dollars in 2007. Herbicides are most widely used in controlling of pests [2]. One of the primary concerns is to minimize harmful effects caused by the target organisms including viruses, bacteria, fungi, and insects [3]. The extensive use of pesticides causes serious environmental concerns, as only 5% or less from the applied pesticides reach the target organisms which resulted in contamination of soil and water bodies (major environmental problem of current age). The periodic use of pesticides makes the situation particularly perturbing. This repetition in the long term necessarily leads to an accumulation of pesticides and their residues in environment, endangering the entire population by their multifaceted toxicity [4]. There is a direct relationship between the contamination of pesticides and their residual detection [5]. In addition to causing toxic effects to humans, there is a high risk of contamination in ecosystem [6]. An enduring threat of volatilization of sprayed pesticides is present that usually hit (directly) nontarget vegetation. This leads towards contamination of air, soil, and nontarget plants [7]. There are chronic threats to human life, caused by long term, low dose exposure to pesticides. It can cause hormonal disruption, diminished intelligence, and reproductive abnormalities [8]. The constant mobility of applied pesticides through leaching, sorption, and volatilization results in contamination of different levels in the environment (Figure 1) [9, 10].

More than one kind of pesticide is applied for the control of different types of pests, as the classification of these substances can be accomplished on the basis of their use, mode of action, and chemical function. These include insecticides for insects control, fungicides against fungi, rodenticide for rodents, and defoliant for leaf harvesting. There are some classes to which pesticides are classified on the basis of their chemical nature, that is, organochlorides, organophosphates, pyrethroids, and so forth. In present age, more than 500 different formulations of pesticides are used mainly in agricultural tricks. These formulations are in general artificially synthesized substances which are nonbiodegradable and enhance environmental toxicity. These nonbiodegradable compounds persist in agricultural fields after application. About three million people are intoxicated per annum as a result of pesticides usage, reported by the World Health Organization (WHO) [6]. The degradation of persistent pesticides is very essential for decontaminating soil and water bodies [13].

The pesticides degradation processes are of different modes, involved in decontamination of various systems in variable efficiency. The rate of degradation of pesticides is influenced by several factors which include chemical structure of pollutants, pH of soil, concentration of hydrogen peroxide, and concentration of iron. The rate of degradation differs as the pathway of this process changes. Acceleration of degradation processes results in decontamination in short span of time. Thus, photocatalytic degradation, biodegradation, ozonation, and photo-Fenton reactions are commonly evaluated for pesticides removal studies [14]. Microorganisms are present on earth as an uncountable number of species. These microbes are very vital for the bioremediation of pesticides. Endosulfan (pesticide) can be removed from environment by applying strains of microbes (*Aspergillus*) [15]. The phenomenon of biotransformations is very common and sometimes very essential for the survival of microorganisms, responsible for biodegradation of applied pesticides. There is a natural balance in between microbial evolution and bioremediation [16]. Biodegradation can be approached via microbes and also augmenting this process by artificial means. This approach to environmental decontamination possesses a number of benefits; for example, there is minimum chance of environmental disruption, economical, and fewer chances of secondary exposure alongside not causing damage to ecosystem [17, 18]. The isolation and characterization of microbial strains capable of degrading pesticides and their residues are of interest for the last two decades. In these microbes bacteria and fungi are the major degraders. Molecular probes can be used for the isolation and identification of degrading potential of microbial strains [18]. Ultimately organic matter decomposes as a result of microbial action. There is mismatch in synthetic and natural occurring pesticides so degradation rate differs in both cases, which is slow in case of synthetic pesticides (due to structural variations and less compatibility with metabolic pathways of applied microbes) [19].

Biodegradation methodology is widely used for the treatment of xenobiotics such as pesticides in soil. It is employed in many countries due to its low cost and being ecofriendly [20, 21]. Conventional approaches like land filling, recycling, and incineration are not very efficient and cost-effective. Different types of toxic intermediates are also formed during these processes [22]. In the present review, different approaches for biological degradation of pesticides have been discussed, in addition to analyzing (on the basis of reported literature) various factors affecting these modes of bioremediation.

## 2. Different Approaches for Biodegradation

Although a number of techniques are available for biodegradation, the ones of utmost importance are discussed here:

- Bacterial degradation.
- Fungal degradation.
- Enzymatic degradation.

### 2.1. Bacterial Degradation

The degradation of pesticides results in the production of carbon dioxide (CO_2_) and water (H_2_O) by the oxidation of parent compounds. The bacterium involves in the degradation process energy intake from these degradation products. The efficiency of degradation process depends upon optimum atmospheric conditions, that is, temperature, pH of soil, moisture contents, and so forth. The modifications of different bacterial specimens via genetic mutations also enhance effectiveness of applied microbes. The biodegradable removal of pesticides has positive effects on the fertility of agricultural soil. Chlorpyrifos has a massive effect on contaminating soil and water bodies. Microbial degradation is very useful for the detoxification of such (chloroorganic) pesticides. The specific genes and enzymes are very critical for the cleavage of specific functional groups of the pesticide. The optimization of environmental conditions and an effective microbial community in the contaminated site is very essential for the degradation of pesticides [23].

There is a vital advantage of microorganism usage for degradation of pesticides. This is due to the diversity, wide distribution, and adaptation of variable metabolic pathways. The gene clusters are involved in microbial degradation. The genetic manipulation and construction of gene engineering bacteria are also used for degradation of pesticides [24]. Microbial strain screening and isolation are very effective for degradation of carbendazim in mineral culture medium. Carbendazim is carbon source for the growth of this strain. The pH range, 5.1–8.1, and temperature range, 25–40°C, are optimum for maximum degradation efficiency, that is, up to 90% in nitrogen atmosphere [25]. Pesticide-degrading bacteria and* Rhizobium meliloti* coating on* Medicago sativa* seeds are effective for repairing soil, polluted by organic phosphorus pesticide. This approach is very efficient, possessing several advantages, that is, rapid soil repairing rate, simple operation, and high treating capability for removal of organic phosphorus pesticide [26, 27].* Sphingobium japonicum* is a strain for degradation of chlorinated pesticides, that is, hexachlorocyclohexane. This strain (*Sphingobium japonicum* LZ-2) can completely decompose lindane 20 mg/L in 10 hours [28]. An aerobic bacterium (*Burkholderia cepacia* strain CH-9) can be used for degradation of imidacloprid and metribuzin. 69% degradation of imidacloprid and 86% degradation of metribuzin can be obtained in 20 days with initial dose of 50 mg/L in mineral salt medium [29]. Bifenthrin (BF) is a synthetic pesticide. It is degraded by pyrethroid bacteria (*Acinetobacter calcoaceticus*). The degradation rate could be achieved up to 56.4% with initial concentration of 100 mg/L with pH range of 6.0–8.0 and 5% inoculation [30].

Streptomycetes strains have enormous applications for degradation of chlorpyrifos (CP) pesticide. The degradation potential of these strains can be evaluated by performing study in agar medium. The pH alterations can affect the efficiency of degradation process [31]. Tert-Bu mercaptan (TMB) undergoes biodegradation in water under aerobic conditions. First-order kinetics are involved in biodegradation process. There is slight increase in rate of reaction by addition of TMB and slight decrease with addition of phenol [32]. Bacterial strains which are capable of degrading methomyl and carbofuran can be studied by high pressure liquid chromatography (HPLC) in biodegradation analysis. Acetonitrile and water were used as mobile phases. The closeness of carbofuran-degrading strains to the genera* Flavobacterium* and* Alcaligenes* and that of methomyl degrading strains to genera* Pseudomonas* and* Alcaligenes* were observable by using 16S rDNA sequence analysis [33, 34]. Photosynthetic bacterium (GJ-22) is capable of degrading cypermethrin (CMP). That CMP degradation by GJ-22 is very productive at 25–35°C and at pH of 7.0. By performing gas chromatography/mass spectrometry (GC-MS), metabolic products are detected. The degradation of CMP proceeds through oxidative or/and through hydrolytic pathways by GJ-22 yielding 5 metabolites [35]. The removal of organochlorine pesticides from soil is performed by microbial applications under optimum environmental conditions. Better results are obtained by addition of potassium humate for increasing concentration of microorganisms [36]. The strain of* Pseudomonas putida* and* Pseudomonas mendocina* has a great capacity of biodegrading permethrin and cypermethrin pesticides. Bioremediation up to 90% can be achieved with the help of these bacterial strains within the period of 15 days [37].

*Acinetobacter* sp. TW and* Sphingomonas* sp. TY strains are novel and very useful for the disposal of tobacco waste in the temperature range of 25–37°C and pH range of 7.0–8.0 [38]. The actinomycete strain HP-S-01 is isolated from activated sludge for its application to degrade deltamethrin. The degradation results in 3-phenoxybenzaldehyde as major hydrolysis product. This strain is highly efficient in degrading bifenthrin, fenvalerate, and fenpropathrin. This process undergoes first-order kinetics and provides an effective tool for bioremediation of environmental contamination from pesticides [39]. Diazinon degrading bacteria utilize it as a source of carbon and phosphorus under different culture conditions. The addition of carbon sources, as glucose or succinate, causes decrease in degradation rate [40]. Biodegradation of profenofos is conducted by bacterial strains isolated by enrichment technique. About 90% concentration of profenofos can be degraded in 90 hours [41].* Paracoccus* sp. strain is applied for the biodegradation studies of pyridine. It was observed that, at the concentration of pyridine <0.9 mg/L, the rate of degradation is higher while at the concentration >0.9 mg/L the rate is lower [42]. A bacterial consortium which degrades tetrachlorvinphos is isolated from agricultural soil. It is composed of six pure strains. The study reveals that these strains have a potential to degrade organophosphate pesticides [43].

Lactic acid bacteria can degrade organophosphorous insecticides by fermentation. Lactic acid bacteria use organophosphate as a source of carbon and phosphorus [44]. An effective and specific method is bacterial degradation of pyrethroid (a pesticide). Highly efficient bacterial strain of* Enterobacter aerogenes* can degrade many other pesticides, that is, bifenthrin, cypermethrin, and so forth [45].* Acinetobacter johnsonii* (MA-19) strain was used for degradation study of organophosphate pesticides, by enrichment culture method. Four additional compounds were added to enhance efficiency, out of which Na succinate was very effective; by increasing its concentration the rate of degradation of malathion increased [46]. The same methodology was applied to degrade para-nitrophenol by* Rhodococcus* bacteria. It is an efficient bacterial decomposition method for para-nitrophenol [47]. Similarly, organophosphate pesticides degradation is carried out by using strains* Bacillus*, Actinobacteria, and L-proteobacteria [48]. Bacterium* Bacillus thuringiensis* is effective in degrading malathion in minimum salt media. With the addition of glucose and yeast, the growth of bacteria increases up to 10^5^-fold which degrades more than 99% malathion within 30 days. Residues were studied by HPLC and GC-MS [49]. Esbiothrin was degraded with much efficiency by immobilized* Acinetobacter* on magnetic polyurethane [50]. By using immobilized bacteria on Ca-alginate gel beads, organophosphate insecticide degradation was studied, along with hydrolyzed products [49].

Cyanobacteria and blue green algae convert fenamiphos into number of its stable, nontoxic components by using cultured technique [51]. Indigenous bacteria degrade sumithion OPs through anaerobic decomposition. They decompose them into CH_4_, N_2_, CO_2_, H_2_S, and so forth [52]. Beans of green coffee can be used for the support and growth of bacteria (*Stenotrophomonas maltophilia*) which degrade DDT and endosulfan. A medium amended with glucose is used as a supplement [53].* Pseudomonas* bacterium can degrade endosulfan. Whenever it bioaccumulates in fishes (*Cyprinus carpio*), it uses endosulfan as a carbon source [54, 55]. Atrazine is degraded by* Pseudomonas* bacteria by two-phase biodegradation (unstable degradation products from first step further degrade to secondary components) [14]. Endosulfan is metabolized into endosulfan sulfate, which is the only product of endosulfan metabolism, by bacterial action. It resulted in 50% degradation of endosulfan within three days [56]. A Gram negative bacterial strain (*Sphingomonas*) possesses high potential for degrading DDT [57]. Microscopic organisms (3 bacterial strains) potentially degrade mefenacet and many other amide pesticides such as propanil and metolachlor by hydrolysis [58].

Different types of pesticides (OPs, chlorinated pesticides, herbicides, and fungicides) are effectively degraded by the fermentation process carried out by* Rhodobacter sphaeroides* [58]. Screened bacteria are highly selective for the degradation of S-enantiomer of methylaxyl compared to its R-enantiomers at comparatively fast rate [59].* Vibrio* and* Shewanella* bacteria can effectively degrade methyl parathion. Its biodegradation mechanism is entirely different from photocatalytic process [60]. Photosynthetic bacteria have capacity to degrade multiple types of pesticides (chlorpyrifos, phoxim, and triazophos) [61].* Ochrobactrum* easily oxidize triazophos into its acidic form. It has the ability to degrade this pesticide up to 95% in crops [62]. Chlorinated pesticides can be degraded by using combination of aerobic-anaerobic decomposition with application of sugar solution. This is one of the very efficient methods for biodegradation of chlorinated pesticides [63]. Allethrin is a pyrethroid insecticide and its degradation is achieved by* Acidomonas* sp. [64]. Eight bacterial strains potentially degrade PCNP pesticide. Better results were obtained when all these strains were collectively used [65]. Two bacteria cad1 and cad2 degrade cadusafos in mineral salt medium with nitrogen (MSMN). They are also able to degrade ethoprophos nematicide completely [66].

Immobilized bacteria have capacity to degrade multiple pesticides (herbicides, fungicides, and carbamates) under different environmental conditions with different flow rates [67]. S-25 strain caused degradation (almost 100%) of 2,4-D organochlorine pesticide at optimum conditions, that is, temperature of 30°C and pH of 7.0 [68]. Aldrin (an organochlorine insecticide) is anaerobically degraded by microorganisms. These microorganisms used extracted yeast as carbon source [69]. Ethion (OPs) is anaerobically degraded by mesophilic bacteria. Other species are also capable of its degradation like* Azospirillum* and* Pseudomonas* [70]. Bacterial consortium, like* Bacillus* sp. and* Chryseobacterium joostei*, was used to compare biodegradation of lindane, methyl parathion, and carbofuran in individual and mixed pesticide enriched cultures by using biokinetic parameters. These bacteria use pesticide in their cometabolic pathways [71]. Psychrotrophic bacterium can degrade Me-parathion. This biodegradation is sensitive to pH and temperature variations [72]. Six genera are able to degrade organochloride pesticides, that is, endosulfan. Different genera have different potential to degrade them, from which* Micrococcus* and* Pseudomonas* were highly active compared to others [73]. Immobilized* Escherichia coli* (a well-known bacterium) could degrade organochlorine insecticide that contains ester bond [74].

The same bacterium is highly efficient in degrading a number of pesticides including BHC, DDT, endosulfan, HCH isomers, and 2,4-D [75–80]. DLL-1 bacterial strain biologically degrades pesticide that is present in soil and plant system [81]. Growth promoting rhizobacteria (GPRB) strains are effective in degrading fungicide and herbicide compared to* Azotobacter* and bacilli. The purpose was to determine the capacity of different bacteria to effectively degrade fungicides and herbicides [82].

### 2.2. Fungal Degradation

Fungi, from natural sources, can be screened out as an effective tool for biodegradation of toxic organic chemicals. A fungal strain* Fusarium verticillioides* is able to use lindane as a carbon and energy source under aerobic conditions. This strain can be isolated from* Agave tequilana* leaves by enrichment techniques. In the presence of limited nitrogen and phosphorus atmosphere, the efficiency in terms of higher degradation is achieved. The environmental factors and concentration of lindane and yeast extract improved the efficiency of the biodegradation process [83, 84]. There is a great potential of fungal strains, that is,* Fusarium oxysporum*,* Lentinula edodes*,* Penicillium brevicompactum*, and* Lecanicillium saksenae*, for the biodegradation of the pesticides like terbuthylazine, difenoconazole, and pendimethalin in batch liquid cultures. These fungal strains are investigated to be valuable as active microorganisms for pesticides degradation [85]. Nonacclimated mixed culture of bacteria and white-rot fungus has applications for biodegradation of aldicarb, atrazine, and alachlor from the liquid phase, respectively. With incubation period of 14 days, mixed culture achieved 47, 98, and 62% removal, respectively. The removal of these pesticides is accompanied by phenomena of biosorption and biodegradation [86].

Methomyl and diazinon (pesticides) are biodegradable with the help of rot fungi isolates from contaminated soil. The optimum temperature for maximum efficiency is 28°C. The rate of degradation is higher by using mixture of fungal strains [87]. Different fungal strains are observed for their degradation ability of DDD pesticide. The accumulation of these strains shows characteristic pattern for degrading process [88]. Endosulfan-degrading, aerobic fungal strains are effective for soil contaminated with organochlorine pesticides. These strains (*Mortierella* sp. strains W8 and Cm1-45) resulted in 50–70% degradation in 28 days at 25°C. The diol formation of endosulfan firstly and then endosulfan lactone conversion take place during degradation. This enhances fertility of agriculture land [89]. On similar basis, there is possibility of degrading mixed insecticides (DDT and chlorpyrifos) by using mixed fungal strains. When low concentration of mixed insecticides was used, the efficiency of degradation is observed to be high. The efficiency is observed in 26.94% and 24.94% degradation of DDT and chlorpyrifos, respectively [90]. Under harsh conditions,* Sphingomonas yanoikuyae* strain can degrade carbamate and pyrethrin (OPs) with high efficiency in enrichment culture method, analyzed by gas chromatography [91]. Salt resistant actinomycete is capable of degrading carbofuran. One of seven actinomycetes,* S. alanosinicus*, is most effective and gives up to 95% degradation. It uses carbofuran as a carbon source and is applicable to saline soils for its efficiency [92].

Water body and soil that are affected by endosulfan can easily be bioremediated by fungal strain (*Aspergillus niger*). The chlorinated pesticide endosulfan is metabolized through various intermediates by this fungus [15]. More than 30 microorganisms are capable of degrading the pesticides, out of which* Gliocladium* genus has maximum activity for selectively degrading carbofuran [93]. Fungus uses chlorpyrifos as a carbon and energy source and causes its rapid degradation. Another fungus, basidiomycetes, degrades chlorpyrifos very effectively [94]. A fungus,* C. elegans*, degrades DEET, an insecticide, into different less toxic metabolites analyzed by HPLC-MS [95]. Phytopathogenic fungi easily degrade herbicides. This fungus easily grows up on organophosphonate herbicides and degrades them [96].* Trichoderma viride* and* T. harzianum* have high potential to degrade pirimicarb. Degradation capacity increases when activated charcoal is added [97].

### 2.3. Enzymatic Degradation

Enzymes produced during different metabolic pathways in plants as well as in microbes present in soil are the key for bioremediation of pesticides. Optimum environmental conditions support fast rate of removal of toxic intermediates. The engineered bacteria were used to produce esterase gene which specifically act on substrate and degrade more than 65% methyl parathion within 3 hours [98]. Carbofuran, an insecticide present in contaminated soil, can be treated with* Paracoccus* sp. YM3, by MSM method, which enzymatically degrades carbofuran into its metabolites which were analyzed by HPLC. This bacterium uses carbofuran as sole source of carbon [99]. Genetically modified* Escherichia coli* enzymatically degrade methyl parathion and many other OPs, that is, PNP, which is detected by HPLC [100].* Micrococcus* sp. has been found to have versatile ability to degrade OPs pesticide like cypermethrin by enzymatic action [101]. Lindane is degraded by fungus* Conidiobolus* through enzyme action. GC-ECD and GC/MS confirm that there is no metabolite; this proved that lindane is completely degraded by this fungus [102]. In a study of atrazine (AT) and alachlor (AL), their degradation by treating them with extracellular enzyme extracted from fungi was determined [103]. FDS-1 strain of* Burkholderia* sp. can degrade nitrophenyl enzymatically at 30°C and pH of 7.0 taken as optimized conditions [104]. Strains of genetically modified bacteria contain enzymes, which potentially can degrade number of pesticides including OPs, carbamates, and pyrethroids [105].

A study revealed that different enzymes specifically degrade different pesticides (OPs) in wheat kernels [106]. Thirty fungal strains were used to investigate degradation rate of Diuron and pyrithiobac-sodium. Results suggested that the highest degrading rate was by ligninolytic enzymes [107].* Enterobacter* enzymatically degrades chlorpyrifos and many other OPs. It degrades them and uses them as carbon and phosphorus source (sole source) [108]. Some Gram negative bacteria have ability to degrade dimethoate. They use it as a sole source of carbon. Bacteria hydrolyze insecticide by using different enzymes, namely, phosphatases and esterases [109]. More than 15 fungal strains were capable of degrading different OPs up to 96% by enzyme catalyzed pathways [110]. Enzymes for the degradation of organochlorinated pesticide are mainly dehydrochlorination enzymes, hydrolytic enzymes, and dehydrogenases. The genes related are Lin family genes with typical functional codes. Further research could be an effective tool for removal of these pesticides [111]. The amino acid sequence of phosphotriesterase mutant is very effective for the application in organophosphorus pesticide degradation [112].

## 3. Conclusion

Decontamination, caused by pesticides usage, of polluted areas is the need of modern age. The applications of conventional means, that is, physicochemical methods, for the degradation of toxic chemicals are not very efficient. These methods are expensive and also not friendly to ecosystem. For the degradation of pesticides and ultimate decontamination of polluted areas, biodegradation is becoming a method of choice. For the removal of hazardous chemicals from environment, the usage of biological agents (bacteria, fungi, and enzymes) is very efficient as they are cost-effective as well as ecofriendly. These biological agents have a potential to decompose pesticides into their less toxic byproducts. There is a need of further study for the investigation of mechanisms of microorganisms and their enzymes during degradation process. The understanding of enzymatic actions, especially concepts related to pesticides mechanism of action, resistance, selectivity, tolerance, and environmental fate, has a vital impact on the knowledge of pesticide science and biological applications.
